# Supplementary material for: In-depth analysis of obesity-associated changes in adipose tissue-derived mesenchymal stromal/stem cells and primary cilia function
Source: Commun Biol. 2025 Oct 13;8:1462. doi: 10.1038/s42003-025-08986-w (PMC12518815; doi:10.1038/s42003-025-08986-w)
Supplement: Supplementary file 1 — Supplementary Information [file 42003_2025_8986_MOESM1_ESM.pdf]

Supplementary Information

Fig. S1

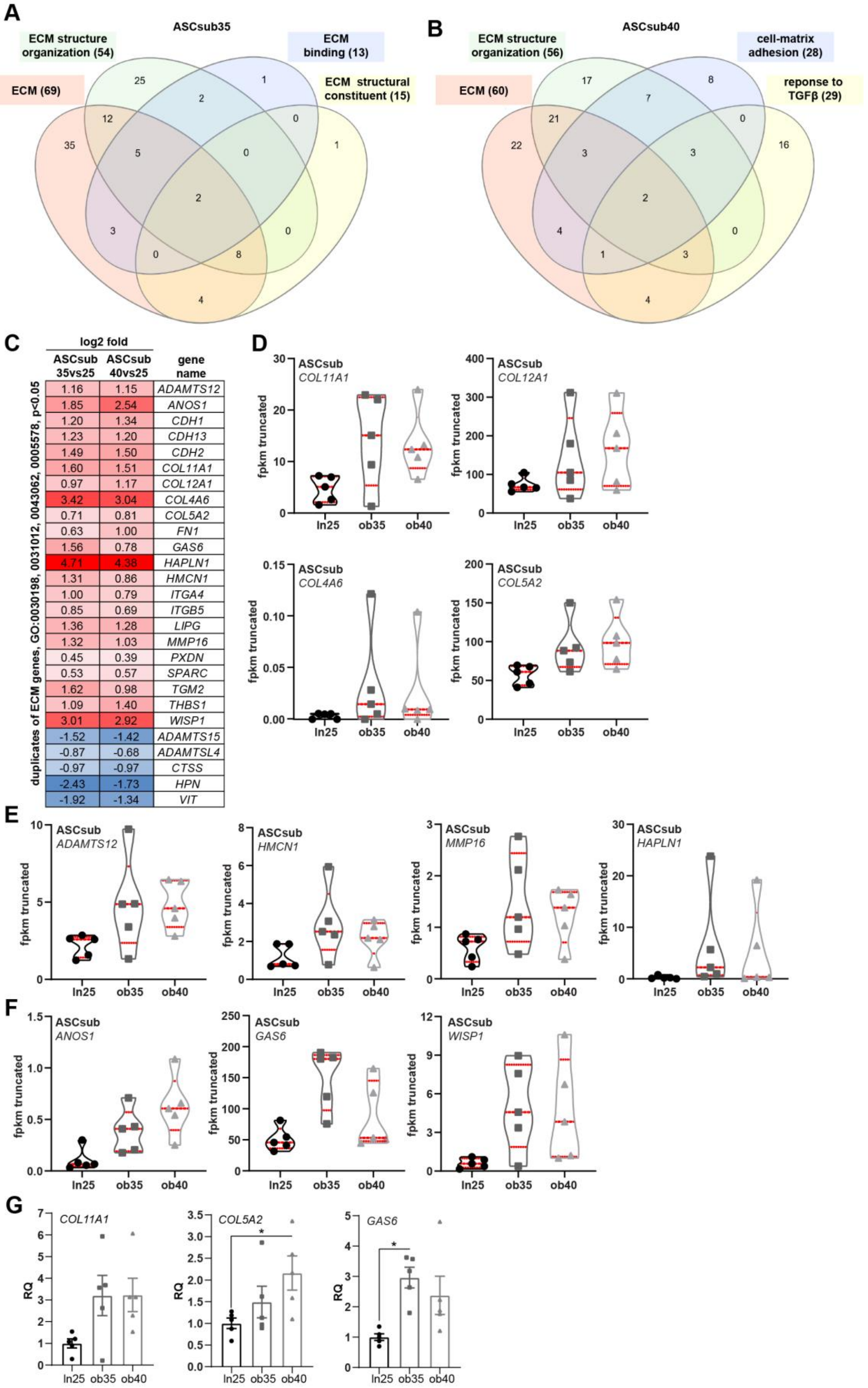

Figure S1: Altered extracellular matrix genes (ECM)

(A and B) Venn diagrams to illustrate the extent of gene overlap among ECM-related GO terms, generated with InteractiVenn[1]. (C) The heatmap depicts significantly ($p < 0.05$) differentially expressed extracellular matrix (ECM) genes with the gene ontology terms GO:0030198, 0031012, 0043062, and 0005578 in both ASC subgroups from donors with obesity, relative to ASCs from lean donors. Significantly enriched genes have a red color code and reduced genes are encoded in blue. Red and blue colored genes are sorted alphabetically. (D-F) Truncated violin plots present selected ECM genes that are differentially expressed. Values reflect the fragments per kilobase per million mapped fragments (fpkm) of genes from the RNA-seq data. Each violin plot displays the median (central dashed line) and quartiles (upper and lower dotted lines). (G) Relative gene levels of *COL11A1* (collagen, type XI, alpha 1), *COL5A2* (collagen, type V, alpha 2), and *GAS6* (growth arrest-specific 6) are shown for lean and obese ASC subgroups (ln25, ob35, and ob40). The results were obtained from five individual samples in each group, and presented as relative quantification (RQ) with SEM. Glyceraldehyde-3-phosphate dehydrogenase (*GAPDH*) was used as endogenous control. Ordinary one-way ANOVA followed by Dunnett’s multiple comparisons test was used to assess statistical significance relative to ln25. $^*p < 0.05$. Abbreviations: COL, collagen; GAS6, growth arrest-specific 6; TGFβ, transforming growth factor-β.

Fig. S2

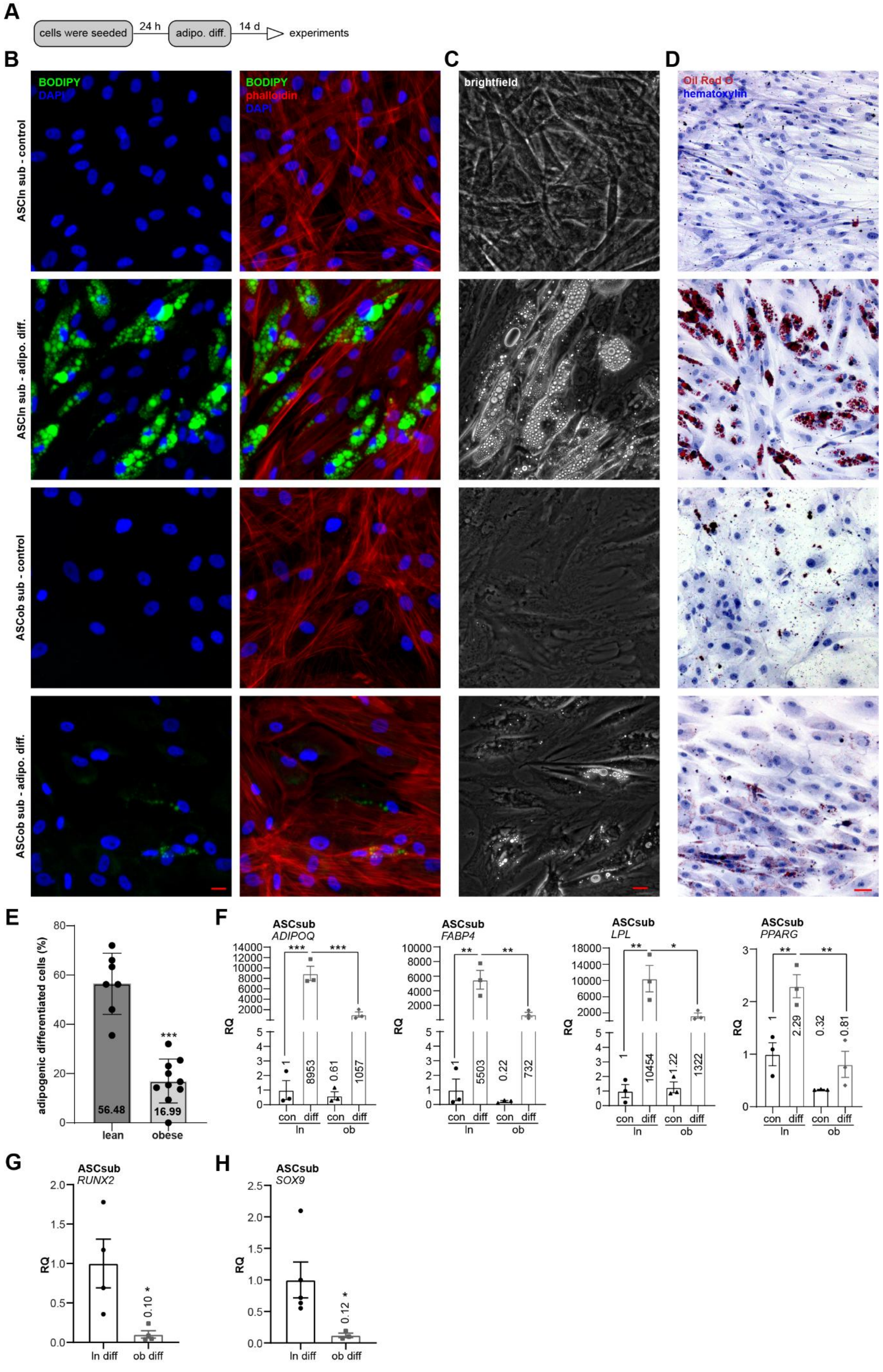

A
cells were seeded
24 h
adipo. diff.
14 d
experiments
B
C
D
BODIPY
DAPI
BODIPY
phalloidin
DAPI
brightfield
Oil Red O
hematoxylin
ASCln sub - control
ASCln sub - adipo. diff.
ASCob sub - control
ASCob sub - adipo. diff.
E
adipogenic differentiated cells (%)
56.48
16.99
***
lean
obese
F
ASCsub
ADIPOQ
ASCsub
FABP4
ASCsub
LPL
ASCsub
PPARG
RQ
1
8953
0.61
1057
1
5503
0.22
732
1
10454
1.22
1322
1
2.29
0.32
0.81
con
diff
ln
ob
G
ASCsub
RUNX2
0.10
ln diff
ob diff
H
ASCsub
SOX9
0.12
ln diff
ob diff

Figure S2: ASCs from patients with obesity have an impaired ability to differentiate into adipocytes

(A) Subcutaneous lean and obese ASCs (ASCln sub and ASCob sub) were induced to adipogenic differentiation (adipo. diff.) for 14 days and their differentiation rates were evaluated. ASCs without differentiation medium were used as controls. (B) ASCs were stained with the lipid droplet dye BODIPY^{TM} (green), the cytoskeleton marker phalloidin (red), and the DNA dye DAPI (blue). Example images are shown. Scale: 20 µm. (C) Brightfield images highlight the lipid accumulation. Scale: 20 µm. (D) The slides were also stained for Oil Red (red) and counterstained with hematoxylin (blue). Scale: 50 µm. (E) Adipogenic differentiated cells with lipid vacuoles were quantified and are presented in percentage. The results of individual ASC subgroups are presented as mean ± SD (n = 3, 414 cells for lean; n = 3, 226 cells for obese). (F) The gene expression of *ADIPOQ* (adiponectin), *FABP4* (fatty acid-binding protein 4), *LPL* (lipoprotein lipase), and *PPARG* (peroxisome proliferator-activated receptor gamma) are shown for undifferentiated (con) and differentiated (diff) ASCs. The results are obtained from three individual patients in each group. (G and H) ASCs were cultured for 21 days under osteogenic and chondrogenic conditions. RT-PCR analysis was performed for the osteogenic key transcription factor *RUNX2* (runt-related transcription factor 2) and the chondrogenic master transcription factor *SOX9* (SRY-box transcription factor 9). For osteogenic differentiation, n = 4 individual samples (lean and obese); for chondrogenic differentiation, n = 5 lean and n = 3 obese individual samples. The results are presented as relative quantification (RQ) with SEM. *GAPDH* was used as the endogenous control. Statistical significance was analyzed with the Student's *t* test (E and G), or, if not Gaussian distributed, with the Mann-Whitney *U* test (H), or the ordinary one-way ANOVA followed by Tukey's multiple comparisons test was used to assess statistical significance between the groups (F). $*p < 0.05$, $**p < 0.01$, $***p < 0.001$.

Fig. S3

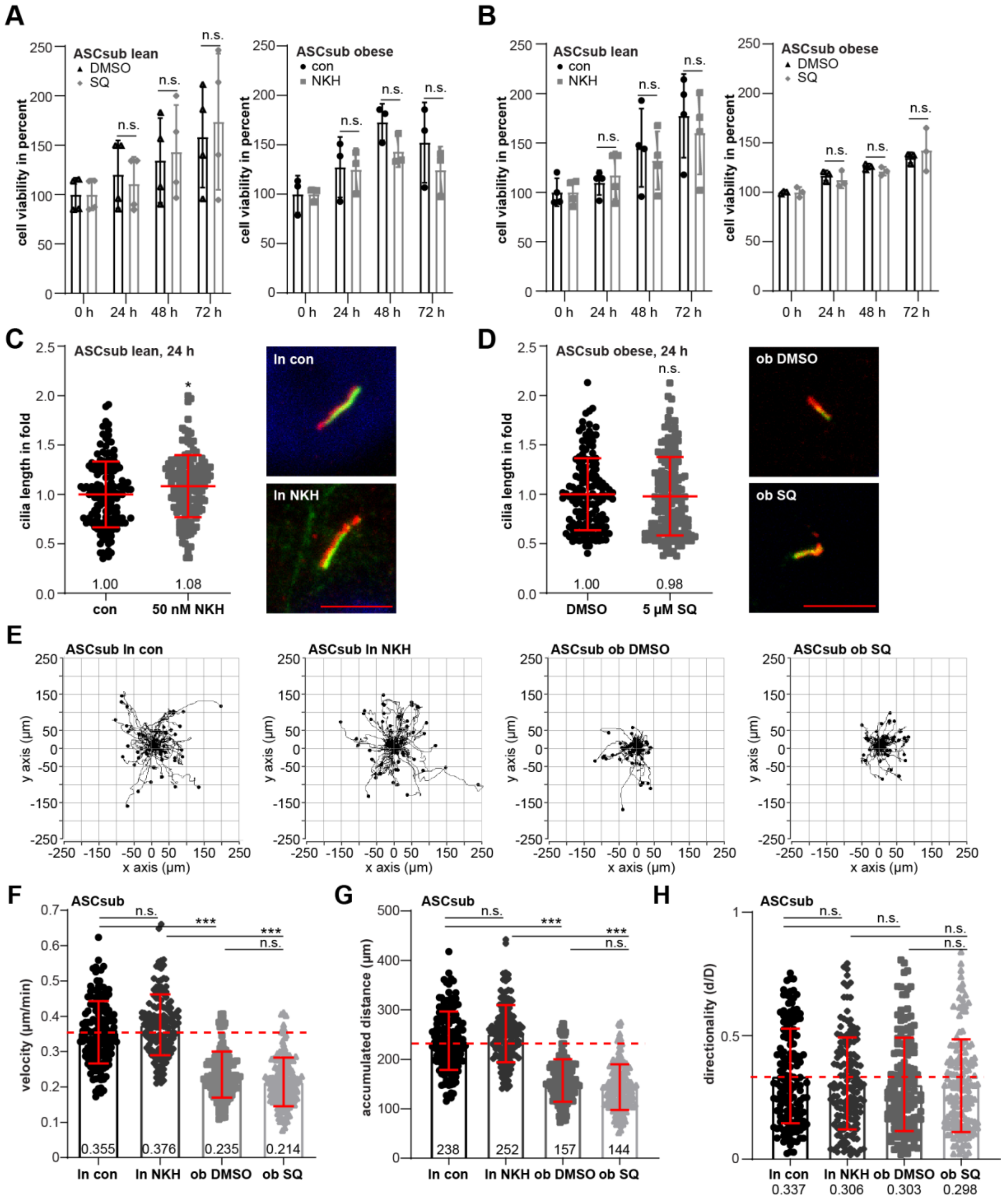

Figure S3: Interfering with ADCY3-cAMP signaling does not influence cell viability

(A and B) Cell viability analyses of ASCsub lean treated with DMSO or the ADCY inhibitor SQ22536 (SQ), and of ASCsub obese treated with the ADCY activator NKH477 (NKH) in (A). In (B), the reverse treatment was applied: ASCsub lean were treated with NKH and

ASCsub obese with DMSO/SQ. Analyses were performed at the indicated time points and presented as mean ± SD. Ordinary one-way ANOVA followed by Sidak's multiple comparisons test was used to assess statistical significance between two groups at each time point (lean: n = 4, obese: n = 3). If variance homogeneity was not met (lean with DMSO/SQ), Welch's ANOVA with Dunnett's T3 multiple comparisons test was used. (C and D) Lean ASCsub were treated with the ADCY activator NKH477 (NKH) (C) or obese ASCsub with its inhibitor SQ22536 (SQ) (D) for 24 h with indicated concentrations. Primary cilia of ASCsub were stained for the ciliary marker acetylated α-tubulin (ace tubulin, green) and ARL13B (ADP-ribosylation factor-like GTPase 13B, red), and DNA (DAPI, blue). Cilium lengths were measured and are presented as scatter dot plots (mean ± SD). Representatives are shown. Scale: 5 μm. Statistical significance was analyzed with the Student's *t* test (C) or with the Mann-Whitney *U* test (D) (n = 3 individual samples, 150 cilia), $*p < 0.05$. (E to H) Analysis of cell motility. ASCs were treated as in (C and D). (E) Representative trajectories are depicted for individual cells (n = 50 cells in each group). The velocity (F), the accumulated distance (G), and the directionality (H) of ASC motility are shown as mean ± SD (n = 3, 150 cells pooled from three independent experiments and individual samples). Statistical significance was analyzed with the Kruskal-Wallis test followed by Dunn's multiple comparison test. $***p < 0.001$. Abbreviations: ADCY3, adenylate cyclase 3DMSO, dimethyl sulfoxide; con, control; n.s., not significant; sub, subcutaneous.

Fig. S4

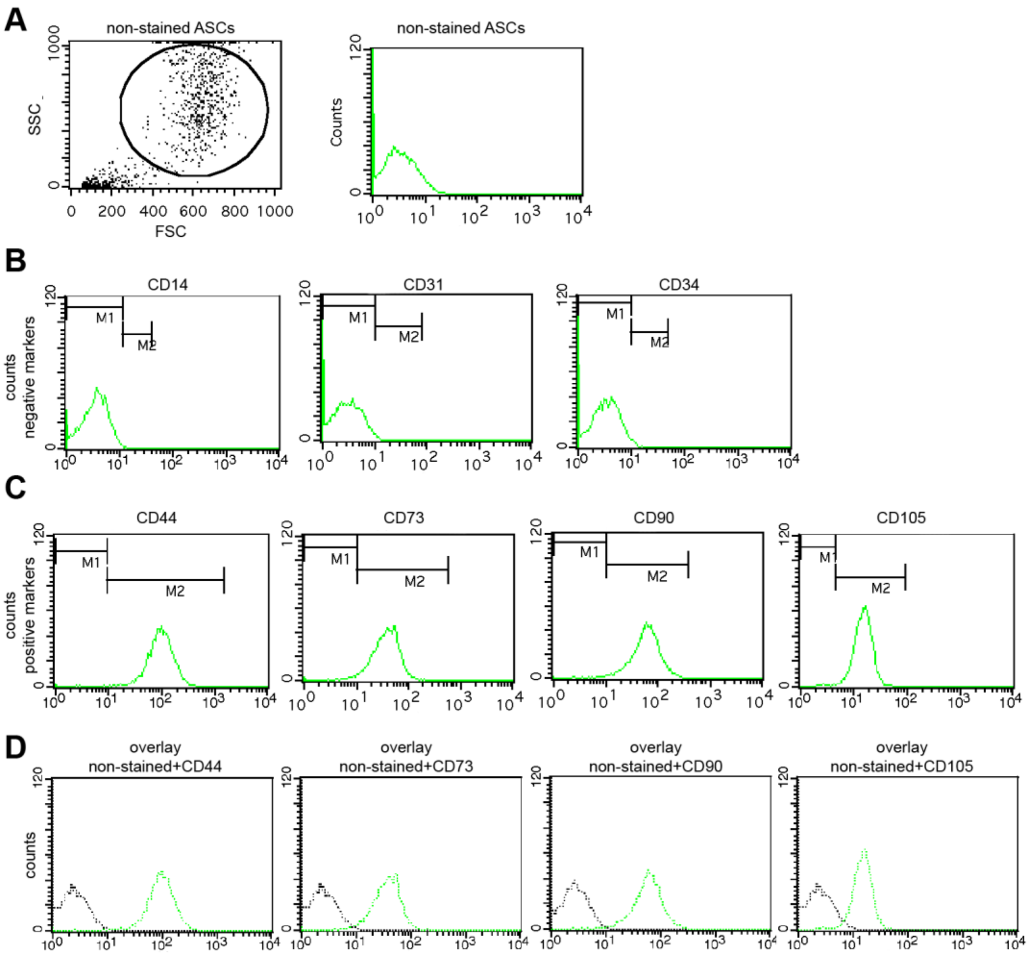

Figure S4: Gating/evaluation strategy for FACS measurements

Representative FACS profiles are shown. (A) Non-stained ASCs were used as negative controls. (B-C) ASCs were characterized for the negative (CD14, CD31, CD34) and positive markers (CD44, CD73, CD90, CD105). (D) Overlay of non-stained ASCs with ASCs stained with positive markers. Abbreviations: CD, cluster of differentiation; FSC, forward scatter; M, marker; SSC, side scatter.

Reference

1 Heberle, H., Meirelles, G. V., da Silva, F. R., Telles, G. P. & Minghim, R. InteractiVenn: a web-based tool for the analysis of sets through Venn diagrams. *BMC Bioinformatics* **16**, 169, doi:10.1186/s12859-015-0611-3 (2015).
