# Supplementary material for: In-depth analysis of obesity-associated changes in adipose tissue-derived mesenchymal stromal/stem cells and primary cilia function
Source: Commun Biol. 2025 Oct 13;8:1462. doi: 10.1038/s42003-025-08986-w (PMC12518815; doi:10.1038/s42003-025-08986-w)
Supplement: Supplementary file 2 — Description of Additional Supplementary Files [file 42003_2025_8986_MOESM2_ESM.pdf]

Description of Additional Supplementary Files

File name- Supplementary Data 1

File description – The source data behind the graphs in the paper are included in the Supplementary Data 1 file
