# Supplementary material for: In-depth analysis of obesity-associated changes in adipose tissue-derived mesenchymal stromal/stem cells and primary cilia function
Source: Commun Biol. 2025 Oct 13;8:1462. doi: 10.1038/s42003-025-08986-w (PMC12518815; doi:10.1038/s42003-025-08986-w)
Supplement: Supplementary file 4 — Reporting Summary [file 42003_2025_8986_MOESM4_ESM.pdf]

nature portfolio

Corresponding author(s): DAPR

Last updated by author(s): Sep 25, 2025

Reporting Summary

Nature Portfolio wishes to improve the reproducibility of the work that we publish. This form provides structure for consistency and transparency in reporting. For further information on Nature Portfolio policies, see our Editorial Policies and the Editorial Policy Checklist.

Statistics

For all statistical analyses, confirm that the following items are present in the figure legend, table legend, main text, or Methods section.

n/a	Confirmed	
☐	☒	The exact sample size (*n*) for each experimental group/condition, given as a discrete number and unit of measurement
☐	☒	A statement on whether measurements were taken from distinct samples or whether the same sample was measured repeatedly
☐	☒	The statistical test(s) used AND whether they are one- or two-sided<br>*Only common tests should be described solely by name; describe more complex techniques in the Methods section.*
☐	☐	A description of all covariates tested
☐	☒	A description of any assumptions or corrections, such as tests of normality and adjustment for multiple comparisons
☐	☒	A full description of the statistical parameters including central tendency (e.g. means) or other basic estimates (e.g. regression coefficient) AND variation (e.g. standard deviation) or associated estimates of uncertainty (e.g. confidence intervals)
☐	☒	For null hypothesis testing, the test statistic (e.g. *F*, *t*, *r*) with confidence intervals, effect sizes, degrees of freedom and *P* value noted<br>*Give P values as exact values whenever suitable.*
☐	☐	For Bayesian analysis, information on the choice of priors and Markov chain Monte Carlo settings
☐	☐	For hierarchical and complex designs, identification of the appropriate level for tests and full reporting of outcomes
☐	☐	Estimates of effect sizes (e.g. Cohen's *d*, Pearson's *r*), indicating how they were calculated

Our web collection on statistics for biologists contains articles on many of the points above.

Software and code

Policy information about availability of computer code

Data collection	No software was used for data collection.
Data analysis	Microsoft®Office®, BD FACStationTM Software v6.0.4 including CellQuest Pro, QuantStudioTM Design & Analysis Software v1.5.2 (Applied Biosystems by Thermo Fisher Scientific), ImageJ 1.49i and the ImageJ plugin Plot Roi Profile (National Institutes of Health, Bethesda, MD, USA), GraphPad Prism 8 or 10 (GraphPad software Inc.), AxioVision Rel 4.8, Chemotaxis and Migration Tool (Ibidi GmbH, Gräfelfing, Germany), web-based tool InteractiVenn and Revigo tool

For manuscripts utilizing custom algorithms or software that are central to the research but not yet described in published literature, software must be made available to editors and reviewers. We strongly encourage code deposition in a community repository (e.g. GitHub). See the Nature Portfolio guidelines for submitting code & software for further information.

Data

Policy information about availability of data

All manuscripts must include a data availability statement. This statement should provide the following information, where applicable:

- Accession codes, unique identifiers, or web links for publicly available datasets
- A description of any restrictions on data availability
- For clinical datasets or third party data, please ensure that the statement adheres to our policy

The RNA sequencing data have been deposited in the NCBI Gene Expression Omnibus (GEO) under accession number GSE308101. The source data behind the

graphs in the paper are included in the Supplementary Data 1 file. All other data supporting the findings of this study are available from the corresponding author upon request.

Research involving human participants, their data, or biological material

Policy information about studies with human participants or human data. See also policy information about sex, gender (identity/presentation), and sexual orientation and race, ethnicity and racism.

Reporting on sex and gender	Sex as a biological variable<br>To investigate the impact of maternal obesity on ASC alterations, subcutaneous adipos tissue samples were collected from women undergoing cesarean sections. This study focused on women since pregnancy occurs in females, but the findings are expected to be relevant to both sexes.
Reporting on race, ethnicity, or other socially relevant groupings	*Please specify the socially constructed or socially relevant categorization variable(s) used in your manuscript and explain why they were used. Please note that such variables should not be used as proxies for other socially constructed/relevant variables (for example, race or ethnicity should not be used as a proxy for socioeconomic status).*<br>*Provide clear definitions of the relevant terms used, how they were provided (by the participants/respondents, the researchers, or third parties), and the method(s) used to classify people into the different categories (e.g. self-report, census or administrative data, social media data, etc.)*<br>*Please provide details about how you controlled for confounding variables in your analyses.*
Population characteristics	*Describe the covariate-relevant population characteristics of the human research participants (e.g. age, genotypic information, past and current diagnosis and treatment categories). If you filled out the behavioural & social sciences study design questions and have nothing to add here, write "See above."*
Recruitment	Women undergoing cesarean sections at term with a body mass index below 25 or above 35 or 40 w/o glucose intolerance.
Ethics oversight	This work was approved by the Ethics Committee of the Johann Wolfgang-Goethe University Hospital Frankfurt (reference number: 375/11, title: Comparison of metabolic pathways in normal and preeclampsia pregnancies, approved newly on 23th November 2021) and informed written consent was obtained from all donors. The Ethics Committee acts in accordance with the Helsinki Declaration. All ethical regulations relevant to human research participants were followed.

Note that full information on the approval of the study protocol must also be provided in the manuscript.

Field-specific reporting

Please select the one below that is the best fit for your research. If you are not sure, read the appropriate sections before making your selection.

☒ Life sciences ☐ Behavioural & social sciences ☐ Ecological, evolutionary & environmental sciences

For a reference copy of the document with all sections, see nature.com/documents/nr-reporting-summary-flat.pdf

Life sciences study design

All studies must disclose on these points even when the disclosure is negative.

Sample size	Most results are based on five individual donors per group or at least three.
Data exclusions	Data point outliers were identified using the Grubbs' test (GraphPath QuickCalcs, San Diego).
Replication	Replications were successful and confirmed previous results: Ritter, A., et al., Characterization of adipose-derived stem cells from subcutaneous and visceral adipose tissues and their function in breast cancer cells. Oncotarget, 2015, or Ritter, A., et al., Primary Cilia Are Dysfunctional in Obese Adipose-Derived Mesenchymal Stem Cells. Stem Cell Reports, 2018.
Randomization	The groups were allocated according to pre-pregnancy BMI.
Blinding	blinded

Reporting for specific materials, systems and methods

We require information from authors about some types of materials, experimental systems and methods used in many studies. Here, indicate whether each material, system or method listed is relevant to your study. If you are not sure if a list item applies to your research, read the appropriate section before selecting a response.

Materials & experimental systems

n/a	Involved in the study
☐	☒ Antibodies
☐	☒ Eukaryotic cell lines
☒	☐ Palaeontology and archaeology
☒	☐ Animals and other organisms
☒	☐ Clinical data
☒	☐ Dual use research of concern
☒	☐ Plants

Methods

n/a	Involved in the study
☒	☐ ChIP-seq
☐	☒ Flow cytometry
☒	☐ MRI-based neuroimaging

Antibodies

Antibodies used

PE-conjugated anti-human cluster of differentiation 44 (CD44) (#130-113-342, clone REA690, lot 5240507442, MACS Miltenyi Biotec, Bergisch Gladbach), PE-conjugated anti-human CD73 (#550257, lot 4149735, BD-Pharmingen, Heidelberg), FITC-CD90 (#11-0909-42, clone eBio5E10, lot 2648893, eBioscience, Frankfurt), PE-conjugated anti-human CD105 (#323206, clone 43A3, lot B418585, BioLegend), PerCP-Cy5.5-conjugated anti-human CD14 (#45-0149-42, clone 61D3, lot 2785933, eBioscience), APC-conjugated anti-human CD31 (#17-0319-42, clone WM-59, lot 2702043, eBioscience), and FITC-conjugated anti-human CD34 (#343504, clone 581, lot B407007, eBioscience), mouse monoclonal antibody targeting acetylated α-tubulin (#T6793, clone 6-11B-1, lot 0000298764, Sigma-Aldrich), rabbit polyclonal antibody against ARL13B (#17711-1-1AP, lot 00142161, Proteintech, Herford), rabbit polyclonal ADCY3 (#19492-1-AP, lot 00046273, Proteintech) and CLUAP1 (#17470-1-AP, lot 00025316, Proteintech)

Validation

CD44, #130-113-342 validated by MACS Miltenyi Biotec for Flow cytometry applications with an epitope competition assay as found here: https://www.miltenyibiotec.com/DE-en/products/cd44-antibody-anti-human-reafinity-rea690.html#Conjugate=PE:size=100-tests-in-200-uL
CD73 #550257 s suitable Flow cytometry (Routinely Tested) according to BD-Pharmingen with 383 product citations
CD90 #11-0909-42, clone eBio5E10 according to eBioscience suitable for Flow Cytometry with 74 publications
CD105 #323206, clone 43A3, BioLegend, for Flow Cytometry, each lot of this antibody is quality control tested by immunofluorescent staining with flow cytometric analysis, 45 product citations
CD14 #45-0149-42, clone 61D3, eBioscience, Flow Cytometry, 68 publications
CD31 #17-0319-42, clone WM-59, eBioscience, Flow Cytometry, ko validated, and 42 publications
CD34 #343504, clone 581, eBioscience, advanced validation, Flow Cytometry in 11 publications
acetylated α-tubulin #T6793, clone 6-11B-1, Sigma-Aldrich, overall 1333 references, suitable for IF
ARL13B #17711-1-1AP, Proteintech, suitable for IF with 631 publications on manufacturer's website
ADCY3 #19492-1-AP, Proteintech for IF, 15 publications, 8 for IFs
CLUAP1 #17470-1-AP, Proteintech, KO validated for WB: T Beyer et al., Mol Cell Proteomics, 2018, CRISPR/Cas9-mediated Genomic Editing of Cluap1/IFT38 Reveals a New Role in Actin Arrangement; suitable for IF

Eukaryotic cell lines

Policy information about cell lines and Sex and Gender in Research

Cell line source(s)

Adipose tissue-derived mesenchymal stromal/stem cells (ASCs) were isolated from women undergoing cesarean sections at term.

Authentication

All ASCs were characterized for the positive (CD44, CD73, CD90, CD105) and the negative markers (CD14, CD31, CD34). Purity of cells was confirmed with positve markers over 95%. And with tri-lineage differentiation capacity.

Mycoplasma contamination

All ASCs were routinely tested for mycoplasma (Minerva Biolabs GmbH).

Commonly misidentified lines
(See ICLAC register)

Name any commonly misidentified cell lines used in the study and provide a rationale for their use.

Plants

Seed stocks

Report on the source of all seed stocks or other plant material used. If applicable, state the seed stock centre and catalogue number. If plant specimens were collected from the field, describe the collection location, date and sampling procedures.

Novel plant genotypes

Describe the methods by which all novel plant genotypes were produced. This includes those generated by transgenic approaches, gene editing, chemical/radiation-based mutagenesis and hybridization. For transgenic lines, describe the transformation method, the number of independent lines analyzed and the generation upon which experiments were performed. For gene-edited lines, describe the editor used, the endogenous sequence targeted for editing, the targeting guide RNA sequence (if applicable) and how the editor was applied.

Authentication

Describe any authentication procedures for each seed stock used or novel genotype generated. Describe any experiments used to assess the effect of a mutation and, where applicable, how potential secondary effects (e.g. second site T-DNA insertions, mosiacism, off-target gene editing) were examined.

Flow Cytometry

Plots

Confirm that:

☐ The axis labels state the marker and fluorochrome used (e.g. CD4-FITC).

☐ The axis scales are clearly visible. Include numbers along axes only for bottom left plot of group (a 'group' is an analysis of identical markers).

☐ All plots are contour plots with outliers or pseudocolor plots.

☒ A numerical value for number of cells or percentage (with statistics) is provided.

Methodology

Sample preparation	Subcutaneous adipose tissue samples were taken from women undergoing cesarean section. Adipose tissue-derived mesenchymal stromal/stem cells (ASCs) were isolated and digested with 1 mg/ml collagenase type I.<br>Cells were trypsinized with 0.25% trypsin (Sigma-Aldrich), harvested, and fixed for 15 min with ice-cold 2% paraformaldehyde. After washing twice with PBS, the cells were stained with cell surface marker antibodies.
Instrument	FACSCaliburTM (BD Biosciences, Heidelberg)
Software	CellQuestPro
Cell population abundance	Non-stained ASCs were used as negative controls. All ASCs were characterized for the positive (CD44, CD73, CD90, CD105) and the negative markers (CD14, CD31, CD34).
Gating strategy	Boundaries at 10^1

☒ Tick this box to confirm that a figure exemplifying the gating strategy is provided in the Supplementary Information.
